# Prediabetes/Diabetes Can Be Screened at the Dental Office by a Low-Cost and Fast Chair-Side/Point-of-Care aMMP-8 Immunotest

**DOI:** 10.3390/diagnostics9040151

**Published:** 2019-10-17

**Authors:** Andreas Grigoriadis, Timo Sorsa, Ismo Räisänen, Pirjo Pärnänen, Taina Tervahartiala, Dimitra Sakellari

**Affiliations:** 1 Department of Preventive Dentistry, Periodontology and Implant Biology, Dental School, Aristotle University of Thessaloniki, 54124 Thessaloniki, Greece; 2 Department of Periodontology, 424 General Army Hospital, 54124 Thessaloniki, Greece; 3 Department of Oral Diseases, Karolinska Institutet, 14104 Huddinge, Sweden; timo.sorsa@helsinki.fi; 4 Department of Oral and Maxillofacial Diseases, Helsinki University and University Hospital, 00014 Helsinki, Finland; ismo.raisanen@helsinki.fi (I.R.); pirjo.parnanen@helsinki.fi (P.P.); taina.tervahartiala@helsinki.fi (T.T.)

**Keywords:** diabetes, HbA1c, MMP-8, dental clinic

## Abstract

Pre-diabetes and diabetes are strongly associated with periodontal disease (gingivitis and periodontitis), and these conditions are known to upregulate aMMP-8 in inflamed gingiva and oral fluids. Thus, it would be feasible to screen for prediabetes and diabetes at the dental office by chairside tests. Chair-side assessment of HbA_1c_ and a quantitative point-of-care (PoC) active matrix metalloproteinase (aMMP)-8 oral rinse immunotest developed for periodontal diseases, were performed on patients (*n* = 69) attending a Periodontology University Clinic who fulfilled the criteria for testing according to the screening questionnaire of the Centers for Disease Control and Prevention, USA. Clinical parameters of periodontal disease were also recorded with an automated probe. Twenty seven-point-five percent of the subjects were found with previously unknown hyperglycemia (HbA1c ≥ 5.7%). There was a statistically-significant positive association between the aMMP-8test and prediabetes (*p* < 0.05, unadjusted and adjusted for BMI and age ≥ 45 years logistic regression models). The dental setting is suitable for opportunistic screening for undiagnosed diabetes and pre-diabetes and point-of-care HbA1c, especially when combined with aMMP-8 assessment by dental professionals, being convenient and effective.

## 1. Introduction

Pre-diabetes and diabetes are associated with an increased rate of initiation, progression, and severity of periodontal disease (gingivitis and periodontitis) in the oral cavity, and this relationship has been established at the pathophysiological and epidemiological level [1,2,3,4]. These conditions are known to upregulate aMMP-8 in inflamed gingiva and oral fluids [5,6]. It has been suggested that screening for prediabetes/diabetes is feasible at dental clinics especially for patients with periodontal disease [7,8,9,10,11]. Therefore, it would be practical and economical to link chair-side/point-of-care (PoC) testing to the diagnostic examination for periodontitis and screening for prediabetes/diabetes at the dentist’s office, since it has been shown that patients meet oral health care professionals on a more regular basis compared to their visits to a physician [12].

## 2. Materials and Methods

Chair-side assessment of HbA_1c_ was performed in a sample of adult patients (n = 69) attending a Periodontology University Clinic. This study was approved by the Ethics Committee of the School of Dentistry, Aristotle University of Thessaloniki, Thessaloniki, Greece (#64, 12/June/2018). All procedures performed in the studies involving human participants were in accordance with the ethical standards of the institutional and/or national research committee and with the 1964 Helsinki declaration and its later amendments or comparable ethical standards and informed consent was obtained from all individual participants included in the study. Patients fulfilled the criteria for testing according to the screening questionnaire of the Centers for Disease Control and Prevention, (CDC) USA [13]. The Cobas Roche b101^®^ diagnostic system was used for the measurement of HbA1c in capillary blood. Matrix metalloproteinase (MMP)-8 (neutrophil collagenase-2) levels in its active form (aMMP-8) in the collected oral rinses were analyzed quantitatively by the chair-side/PoCPerioSafe^®^ immunotest accompanied by the digital reader ORALyzer^®^ according to the manufacturer’s instructions [14,15]. For verification, oral rinses were analyzed by Western immunoblots utilizing specific polyclonal antibodies for MMP-1, -8, and -13 [16]. Body mass index (BMI), age, level of education, and smoking were also recorded. Clinical periodontal and oral health parameters including probing depth, clinical attachment loss, bleeding on probing (BOP), and presence/absence of plaque were assessed for six surfaces of each tooth, excluding third molars, with an automated probe (Florida probe, Florida Probe Corporation, Gainesville, FL, USA).

### Statistical Methods

Statistical analyses were performed with the SPSS Base 25.0. Statistical Software Package (SPSS Inc., Chicago, IL, USA). Association between the aMMP-8 PoC test (PerioSafe^®^/ORALyzer^®^) and prediabetes was assessed by logistic regression analysis (both unadjusted and adjusted for BMI and age ≥ 45 years old). The receiver operating curve (ROC) and area under the ROC curve (AUC) were used to offer an example for the ability of the aMMP-8 PoC test, in combination with some prediabetes risk factors, to classify patients with and without prediabetes. Statistical significance was determined with *p*-values ≤ 0.05.

## 3. Results

Patient characteristics and periodontal parameters are presented in Table 1. Twenty seven-point-five percent of the subjects tested were found to have previously unknown hyperglycemia (HbA1c ≥ 5.7%). There was a statistically-significant positive association between the aMMP-8 PoC test (PerioSafe^®^/ORALyzer^®^) and prediabetes (defined here as HbA1c ≥ 5.7%) (*p* < 0.05), but not between BOP and prediabetes (*p* > 0.05) (Table 2). This was the result from both unadjusted and adjusted (for BMI and age ≥ 45 years) logistic regression models; BMI and age ≥ 45 years are known risk factors for prediabetes. A significant positive association between the aMMP-8 PoC test and periodontal condition (Stage I/II, Grade A–C) (*p* < 0.05), according to the 2018 classification of periodontal diseases [3] and also between BOP and periodontal condition (Stage I/II, Grade A–C) (*p* < 0.01) was also observed (Table 1). This was the case in both unadjusted and adjusted (for smoking, gender, age, and education) logistic regression models. Using BMI and age ≥ 45 years in a logistic regression model produced AUC = 0.683 (*p* = 0.020) in ROC analysis, while adding the aMMP-8 PoC test into that model produced AUC = 0.759 (*p* = 0.001)

**Figure 1:**
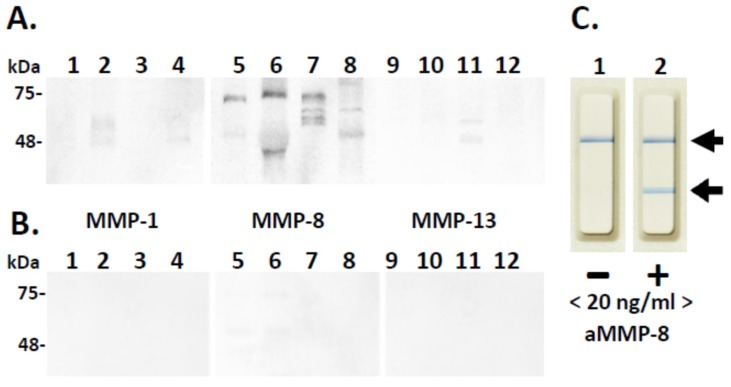
Western immunoblot analysis of diabetic periodontitis-affected (**A**) and systemically- and periodontally-healthy (**B**). Oral rinses for MMP-1 (Lanes 1–4), MMP-8 (Lanes 5–8), and MMP-13 (Lanes 9–12). Mobilities of the molecular weight markers (kDa) are indicated on the left. Negative (−, <20 ng/mL aMMP-8, Lane 1) and positive (+, ≥20 ng/mL aMMP-8, Lane 2) chair-side (PoC) lateral flowimmunotest outcomes are indicated by arrows on the right (**C**).

Moreover, we found a significant association between periodontal condition (Stage I/II, Grade A–C) and prediabetes in unadjusted and some of the adjusted logistic regression models (Table 1). This suggests that prediabetes may have a negative effect on periodontal condition and vice versa. Western immunoblot and aMMP-8 oral rinse immunotest analysis utilizing independent and specific polyclonal and monoclonal antibodies for aMMP-8 disclosed and verified that MMP-8 was elevated, activated, and fragmented in the diabetic mouth rinse samples (Figure 1). Hardly any MMP-1 or MMP-13 immunoreactivities were detected in these diabetic oral rinse samples (Figure 1). No MMP-1, -8, or -13 immunoreactivities could be detected in the systemically- and periodontally-healthy control oral rinses (Figure 1).

## 4. Discussion

Periodontal disease in the form of gingivitis and periodontitis is widespread and significantly affects both individual welfare and healthcare systems. It is also important to report that a link between the presence of periodontitis and negative consequences for general health has also emerged. Extensive epidemiological data have shown that periodontal disease increases the risk of poor glycemic control in patients with type 2 diabetes mellitus (T2DM), as well as diabetes complications and associated morbidity, due to the release of bacterial products and inflammatory mediators from periodontal pockets into the bloodstream [1,2,17]. Findings of the present study suggest that screening at the dental office for prediabetes/diabetes based on a validated questionnaire (such as the one suggested by CDC) is greatly enhanced by the aMMP-8 chair-side/PoC testing. Therefore, it is suggested that this chairside test has an important value in assisting both oral and medical health care professionals in identifying patients that are not only at risk of periodontitis, but also at risk of developing T2DMor having an existing diabetes condition. This low-cost translational PoC-diagnostic procedure can be done on-line chair-side visually and/or quantitatively in 5 min at the dentist’s office. Dental patients who fulfill questionnaire-based validated criteria for diabetes will benefit from the aMMP-8 PoC test when used in combination with other known risk factors.

In line with previous reports, this study provides further supporting evidence that the dental setting is ideal for opportunistic screening for diabetes and that point-of-care HbA1c, especially when combined with aMMP-8 assessment by dental professionals, is convenient and effective at identifying undiagnosed diabetes and pre-diabetes.

## 5. Conclusions

Findings from the present study translationally and economically linked medical and dental professionals regarding global screening for both prediabetes/diabetes and periodontitis. Further studies, with larger subject samples, are required in order to establish this approach in clinical settings.

## Figures and Tables

**Table 1:** Patient characteristics and periodontal parameters (n = 69).

	Prediabetes HbA1c ≥ 5.7			Periodontal Condition		
	HbA1c < 5.7	HbA1c ≥ 5.7	*p*-Value	Healthy	Stage I/II, Grade A–C	*p*-Value
Sex (N)			0.387 ^a^			0.907 ^a^
Women	18	9		8	19	
Men	32	10		13	29	
Age mean (SD)	48.94 (11.59)	56.37 (11.91)	0.036 ^b^ 0.027 ^c^	46.29 (14.14)	53.13 (10.51)	0.045 ^b^ 0.056 ^c^
Education level (N)						
Elementary	1	1	0.038 ^a^	0	2	<0.001 ^a^
				1	27	
Middle	15	13		7	2	
Post graduate studies	8	1		0	4	
				13	13	
Technical school	4	0				
University	22	4				
Annual dental visit (N)			0.344 ^a^			0.945 ^a^
Yes	30	9		12	27	
No	20	10		9	21	
Prediabetes HbA1c ≥5.7 (N)			–			0.027 ^a^
Yes	0	19		2	17	
No	50	0		19	31	
HbA1c (mean, SD)	5.15 (0.33)	6.27 (0.90)	<0.001 ^b^ <0.001 ^c^	5.30 (0.31)	5.53 (0.86)	0.287 ^b^ 0.099 ^c^
eAG (mean, SD)	105.26 (5.44)	133.32 (26.00)	<0.001 ^b^ <0.001 ^c^	107.17 (8.12)	118.05 (23.32)	0.049 ^b^ 0.012 ^c^
Age ≥ 45 years (N)			0.431 ^a^			0.212 ^a^
Yes	34	15		13	36	
No	15	4		8	11	
BMI (mean, SD)	29.14 (4.03)	32.21 (5.69)	0.046 ^b^ 0.041 ^c^	29.25 (4.12)	30.33 (4.97)	0.525 ^b^ 0.352 ^c^
Smoking (N)			0.849 ^a^			0.096 ^a^
Yes	17	6		4	19	
No	33	13		17	29	
Toothcount (mean, SD)	25.46 (2.84)	23.89 (3.28)	0.029 ^b^ 0.077 ^c^	26.53 (1.86)	24.38 (3.22)	0.004 ^b^ 0.001 ^c^
4-mm pocket count (mean, SD)	37.50 (38.17)	52.79 (41.12)	0.182 ^b^ 0.170 ^c^	6.00 (6.53)	57.33 (37.39)	<0.001 ^b^ <0.001 ^c^
5-mm pocket count (mean, SD)	19.58 (29.50)	28.53 (30.06)	0.081 ^b^ 0.275 ^c^	0.95 (1.99)	31.27 (31.46)	<0.001 ^b^ <0.001 ^c^
≥6-mm pocket count (mean, SD)	7.28 (13.17)	9.84 (14.63)	0.119 ^b^ 0.510 ^c^	0.24 (0.70)	11.38 (15.04)	<0.001 ^b^ <0.001 ^c^
BOP (%) (mean, SD)	62.66 (22.96)	64.92 (24.80)	0.762 ^b^ 0.732 ^c^	48.03 (27.86)	69.95 (17.52)	<0.001 ^b^ 0.003 ^c^
Plaque (%) (mean, SD)	61.97 (23.73)	57.70 (25.54)	0.406 ^b^ 0.533 ^c^	48.91 (25.02)	65.99 (22.02)	0.010 ^b^ 0.011 ^c^

N: frequency; SD: standard deviation; BMI: body mass index; BOP: bleeding on probing. ^a^ Mann–Whitney U-test (exact, 2-sided). ^b^ Pearson Chi-squared test (asymptotic, 2-sided). ^c^ Welch *t*-test.

**Table 2:** Unadjusted odds ratios (OR) from logistic regression analysis results showing the association between the active MMP-8 (aMMP-8) point-of-care test (PerioSafe^®^/ORALyzer^®^)and prediabetes/periodontal condition (Stage I/II, Grade A–C) [2] and between bleeding on probing (BOP %) and prediabetes/periodontal condition (Stage I/II, Grade A–C).

	Prediabetes HbA_1c_ ≥ 5.7		Periodontal Condition (Stage I/II, Grade A–C)				
	Unadjusted	Adjusted (BMI, Age ≥ 45 years)	Unadjusted	Adjusted (Smoking)	Adjusted (Smoking, Gender)	Adjusted (Smoking, Gender, Age)	Adjusted (Smoking, Gender, Age, Education)
	OR (CI 95%), *p*-value	OR (CI 95%), *p*-value	OR (CI 95%), *p*-value	OR (CI 95%), *p*-value	OR (CI 95%), *p*-value	OR (CI 95%), *p*-value	OR (CI 95%), *p*-value
aMMP-8 (PerioSafe-ORALyzer^®^)	1.036 (1.007–1.066), 0.016	1.035 (1.003–1.067), 0.031	1.101 (1.012–1.196), 0.025	1.102 (1.013–1.199), 0.024	1.103 (1.013–1.201), 0.024	1.109 (1.015–1.213), 0.023	1.119 (1.005–1.246), 0.040
BOP%	1.004 (0.981–1.028), 0.717	1.007 (0.982–1.032), 0.584	1.049 (1.019–1.079), 0.001	1.047 (1.017–1.078), 0.002	1.047 (1.017–1.078), 0.002	1.046 (1.014–1.080), 0.005	1.044 (1.006–1.084), 0.022
HbA1c ≥ 5.7%			5.210 (1.081–25.104), 0.040	5.666 (1.148–27.957), 0.033	6.046 (1.188–30.763), 0.030	4.550 (0.863–23.993), 0.074	3.396 (0.393–29.356), 0.267
